# Does the Composition of Breast Milk in the First Week Postpartum Differ Due to Maternal Factors or Neonatal Birth Weight and Percent Fat Body Mass?

**DOI:** 10.3390/nu16193310

**Published:** 2024-09-30

**Authors:** Karolina Karcz, Paulina Gaweł, Barbara Królak-Olejnik

**Affiliations:** Department of Neonatology, Wroclaw Medical University, 50-367 Wrocław, Poland; pszczygiol@usk.wroc.pl (P.G.); barbara.krolak-olejnik@umw.edu.pl (B.K.-O.)

**Keywords:** breast milk, human milk, milk composition, body composition, gestational diabetes

## Abstract

Background: The composition of breast milk is dependent on numerous factors. However, the precise impact of maternal health conditions on breast milk composition remains to be fully elucidated. Similarly, there is a paucity of evidence regarding the correlation between neonatal body composition and human milk. The objective of the study was to evaluate the macronutrient composition of breast milk collected during the first week postpartum from mothers with gestational diabetes and healthy mothers in terms of selected maternal and neonatal factors. Methods: *n* = 70 breast milk samples were analyzed for fat, protein, carbohydrate, dry matter, true protein, and energy. The results were evaluated in terms of selected maternal factors, as well as neonatal birth weight (BW) and percent fat body mass (%FBM), which was assessed with a bioimpedance method. Results: Energy of breast milk in the study group was as follows: median 55.5 kcal/dL in GDM G1, median 55.5 kcal/dL in GDM G2, and median 65.0 kcal/dL in non-GDM, which differed significantly in Kruskal–Wallis ANOVA. Neonatal %FBM, but not BW, was found to be significantly related to concentrations of protein, true protein and dry matter. Maternal gestational weight gain, history of hypothyroidism, and classification by study group were identified as factors affecting both breast milk and neonatal body composition. Conclusions: The composition of breast milk in the initial week following childbirth is predominantly determined by maternal factors. The relationship between selected macronutrients and neonatal percent fat body mass was found to be weak, yet the significance of this finding is unclear. Further research is required to ascertain the influence of maternal milk composition on early infantile nutritional programming.

## 1. Introduction

It is already known that the composition of breast milk depends on, for example, the duration of lactation, genetic and environmental factors, and to some extent on the mother’s diet and lifestyle [1,2,3]. The exact influence of maternal health conditions on the average levels of certain nutrients in breast milk is not yet fully understood [4]. A systematic review by Wu suggested that gestational diabetes may alter the composition of human milk, but the small number of studies and the substantial heterogeneity in their concepts and methodologies make it very hard to come to a firm and consistent conclusion [5]. Regardless of the disparity in several methodological aspects of the evaluated studies, it was noted by de Oliveira Lopes et al., that thyroid diseases cause nutritional changes in the human milk long-term, especially in protein content [6]. The results of a review by Hashemi Javaheri et al. confirm that the maternal Body Mass Index alters the profile of fatty acids in human milk, however, varying results were noted for other components of human milk [7].

Exclusive breastfeeding ensures the best nutrition for infants in the first months of their lives. Mothers produce milk that contains all the proteins, fats, carbohydrates, vitamins, and minerals a baby needs. The benefits of breastfeeding have been well documented. Breast milk contains antibodies and other compounds that boost a baby’s immune system, providing protection against infections. It can help reduce the risk of obesity, type 2 diabetes, and certain childhood cancers later in life. Breastfeeding has also been linked to improved cognitive development in children and higher IQ [8,9,10]. To meet the infant’s needs for growth and development, the mother’s milk production must be adequate. In fact, it appears that the volume of milk consumed by the infant is a more important determinant of the infant’s growth than the energetic value of the milk. Surprisingly, concentrations of milk components are not often correlated with infant growth. However, more commonly, infant intakes (or doses) of specific nutrients have been found to follow the evolution of infant body composition during the first year of life [11,12,13].

In addition to its impact on milk composition, maternal health status and the presence of concomitant diseases have been demonstrated to influence fetal growth and the anthropometric measurements and body composition of newborns. This topic has been previously addressed by the authors in another article [14]. Although a substantial body of research has examined the impact of maternal factors on milk composition and neonatal anthropometric parameters, there is a notable scarcity of data exploring the relationship between initial neonatal body composition and initial breast milk composition.

The aim of the study was to evaluate the macronutrient composition of breast milk collected during the first week after delivery from mothers diagnosed with gestational diabetes (GDM), both treated with diet (GDM G1) or insulin (GDM G2), compared to samples from healthy non-diabetic mothers (non-GDM). The macronutrient content of breast milk was also evaluated in relation to neonatal birth weight and percent body fat, as well as other maternal factors previously recognized as predictors of neonatal body composition [14].

## 2. Materials and Methods

### 2.1. Study Design and Material

This was a prospective observational case-control study. The study was started in 2020 and was interrupted during the COVID-19 pandemic because of insufficient enrollment. In 2023, the study was restarted.

The detailed data concerning mothers and their newborns, as well as eligibility criteria, were presented elsewhere [14]. The general participation criteria included mothers aged between 18 and 45 years who had delivered an infant from a single pregnancy after completing the 34th week of gestation (at least 35 + 0/7 weeks). In addition, the following criteria were considered: the condition of the newborn, the practice of exclusive or predominant breastfeeding, as well as a lack of critical conditions in both mother and the newborn infant (maternal substance abuse, maternal lack of medical care during gestation, recognized fetal growth restriction, and severe congenital defects).

The current article presents the preliminary results of breast milk composition in the first week postpartum in relation to selected maternal characteristics, as well as neonatal body parameters—birth weight (BW) and percent fat body mass (%FBM)—in *n* = 70 pairs of enrolled mothers and their newborns.

The dataset consisted of the results of the newborn’s anthropometric measurements, along with clinical data pertaining to the course of the mother’s pregnancy, gestational weight changes, and her pregestational medical history. This was complemented by information on the childbirth process and the postpartum period, as reported by the mother. The data were collected from the mother during the newborn’s postnatal hospitalization in the Department of Neonatology.

In general, the study group included neonates in the first week of life—all were born at term (>/37 weeks of gestation) and had an Apgar score > 7 points at 1 min of age. None of them had significant congenital defects or metabolic disorders.

In consideration of the maternal medical interview, the primary findings were as follows. In regard to maternal thyroid status, *n* = 20 women were diagnosed with chronic thyroiditis, while *n* = 13 developed gestational hypothyroidism. All of these women were successfully treated with levothyroxine. In the analysis of hypertension, it was determined that the condition was chronic in six mothers and induced by pregnancy in eight mothers. All women were treated with methyldopa. Nicotine use before pregnancy was identified in *n* = 22 mothers, and all of them reported quitting smoking before conception. The majority of the mothers (57%) had a normal body mass index (BMI) prior to the pregnancy. In the remaining cases, the BMI was above the upper limits.

The participants were classified into three primary study groups: GDM G1, GDM G2, and non-GDM. Concerning the mothers, *n* = 50 were diagnosed with gestational diabetes, of whom *n* = 21 were treated with diet and physical activity (GDM G1) and *n* = 29 required insulin therapy (GDM G2). All of the GDM mothers received regular medical care and appropriate treatment in accordance with standard clinical practice, which resulted in a satisfactory glycemic control. *n* = 20 mothers did not develop any glucose metabolism disorders in pregnancy (non-GDM). For further details, please check the reference article [14].

### 2.2. Body Composition Analysis

Neonatal body composition was assessed using a non-invasive method of bioimpedance analysis (BIA), with measurements made using the Body Composition Monitor (BCM, Fresenius Medical Care, Bad Homburg, Germany) and special disposable electrodes BCM-FMC (<25 kg). This was done according to the instructions provided by the manufacturer, as described in the previous paper [14]. No significant differences were found in birth weight—BW (F (2, 67) = 2.633, *p* > 0.05), and body fat—FBM% (F (2, 67) = 1.610, *p* > 0.05). For more results, please refer to the primary article [14].

### 2.3. Breast Milk Analysis

The authors and participating mothers identified breastfeeding as a priority. All mothers were provided with adequate support from a lactation counselor. The mothers consented to participate in the study on the condition that the primary objective would be to feed their newborn infants. In line with this rationale, the milk samples provided for analysis were obtained subsequent to the infant’s feeding, thus representing hindmilk. All milk samples were collected over the course of several days following the maternal and neonatal postpartum hospitalization. The mothers declined to provide multiple milk samples for research purposes. Moreover, they declined to participate in post-discharge follow-up and, in some cases, ceased breastfeeding entirely.

Breast milk samples were collected from mothers up to 7 days postpartum, with a mean of 3.4 (SD ± 1.0) days. All samples were collected using a breast pump in the morning after the first feeding of the newborn. A total of 5 mL of the pumped milk was allocated for compositional analysis. The remaining volume was returned to the mother. Milk samples (5 mL) were refrigerated immediately after collection, portioned, and frozen (−80 °C) until analysis. Macronutrient content was measured using the Human Milk Analyzer (HMA), Miris, Uppsala, Sweden. Samples collected from all participants were analyzed for total fat, total protein, carbohydrate, dry matter, true protein and energy.

### 2.4. Software

Microsoft Excel for Microsoft 365 version 2409 of compilation 16.0.18025.20030 (Microsoft, Redmond, WA, USA), Statistica 13.3 (StatSoft, Inc., Tulsa, OK, USA), and R Version 3.6.2 (R Core Team, 2013. R Foundation for Statistical Computing, Vienna, Austria. URL http://www.R-project.org/; accessed on 1 March 2021; packages: ‘stats’, ‘mclust’, ‘boxplot’, ‘MASS’) were applied.

### 2.5. Statistics

The level of significance for the statistical analysis was set to be α = 0.05, *p* < 0.05 was defined as significant. Data were reported as mean and standard deviation (SD), or median and interquartile range (IQR), or number of cases (*n*) and percentage when applicable. Depending on the type of data and their distribution, comparisons of data between study groups were made using one-way ANOVA, or the Kruskal–Wallis test. The Shapiro–Wilk test was used for the assessment of whether a dataset had a normal distribution. Levene’s test was performed to assess equality of variances for variables. The effect of selected neonatal factors on breast milk composition was assessed by univariate regression (generalized linear model). Cluster analysis was conducted as described in another manuscript [14]. To summarize, the processing took place as follows. Univariate regression (generalized linear model) was used to assess the effect of selected maternal factors on the studied neonatal anthropometric parameters and body composition. As a result, study group membership, maternal history of hypothyroidism, and maternal weight gain during pregnancy were selected as the best predictors of neonatal anthropometrics and body composition. The next step was cluster analysis with the Marczewski–Steinhaus (M–S) taxonomic approach and dendrogram construction [15]. The verification of the taxonomic method was carried out with the Expectation Maximization (E-M) algorithm [16]. The present manuscript presents a comparative analysis of the results for breast milk composition, with a focus on the clusters identified in the previous study [14].

### 2.6. Ethics

The study was conducted according to the tenets of the Declaration of Helsinki and was approved by the Bioethics Committee of the Medical University of Wroclaw, Poland (protocol code KB 950/2022, approved 21 December 2022, as a continuation of KB 773/2019, approved 25 November 2019, KB 35/2020, approved 16 January 2020, 407/2020, approved 23 June 2020). Written informed consent was obtained from the mothers included in the study groups. The current study was registered in the Clinical Trials Registry (https://clinicaltrials.gov/, accessed on 18 May 2021) and assigned trial number NCT04937348.

## 3. Results

### 3.1. Demographic and Medical Data in the Primary Study Groups

All of the mothers were of Polish nationality. The median gestational age at delivery was 39.0 weeks (range 47–41 weeks), with no statistically significant difference between the study groups (*p* > 0.05). Additionally, the frequency of cesarean sections and the ratio of male to female infants were comparable (*p* > 0.05). For further details, please refer to the aforementioned source article [14].

There were no statistically significant differences between the study groups in terms of the mean birth weight (3.23 kg), length (53.0 cm), and head circumference (34.5 cm) of the newborn infants (*p* > 0.05).

The mothers in all study groups were of comparable age (mean 32.7 years), gravidity (median 2.0), and parity (median 2.0), *p* > 0.05. All study groups had a similar (*p* > 0.05) number of hypothyroidism (chronic or gestational), as well as hypertension (chronic or pregnancy induced). However, they differed in pregestational BMI (median 23.34 kg/m^2^ (IQR 3.48) in GDM G1, median 28.04 kg/m^2^ (IQR 6.8) in GDM G2, median 22.96 kg/m^2^ (IQR2.83) in non-GDM, *p* < 0.05), and gestational weight gain (GWG) (mean 10.2 kg (SD 3.5) in GDM G1, mean 9.2 (SD 5.4) in GDM G2, mean 16.2 kg (SD 5.4) in non-GDM, *p* < 0.05).

### 3.2. Analysis of Breast Milk Composition

**Figure 1:**
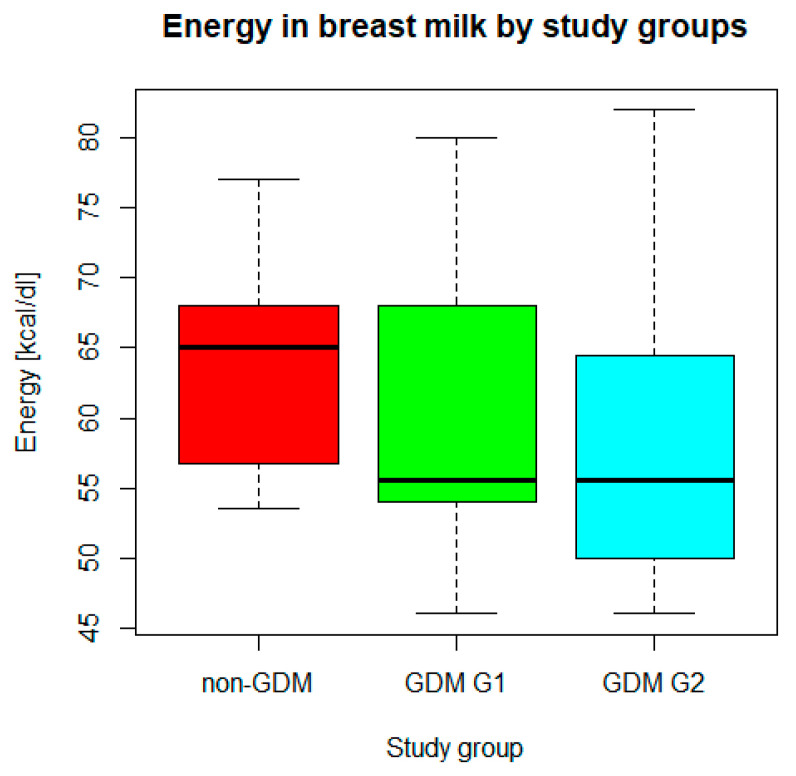
Energy in breast milk in GDM G1, GDM G2, and non-GDM groups. Legend: central line—median, bars—interquartile range (IQR), whiskers of the box plots—range of values/most extreme data.

**Figure 2:**
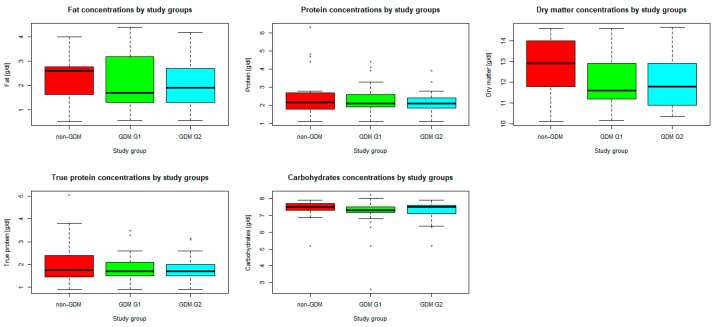
Differences in concentrations of the analyzed macronutrients by study groups. Legend: central line—median, bars—interquartile range (IQR), whiskers of the box plots—range of values/most extreme data, dots—outliers.

*n* = 70 mothers whose newborns underwent body composition analysis had their breast milk analyzed for macronutrient composition. In the study group, mean (SD) concentration of fat was 2.2 (1.02) g/dL, whereas mean (SD) concentration of carbohydrates was 7.2 (0.82) g/dL. Mean (SD) energy was measured as 60.3 (9.6) kcal/dL. The medians (IQRs) of total protein, true protein, and dry matter were 2.1 (1.8–2.5) g/dL, 1.7 (1.5–2.1) g/dL, and 12.0 (11.2–13.3) g/dL, respectively. Taking into account the comparison between the study groups (GDM G1, GDM G2, and non-GDM), only the difference in energy (Figure 1) was found significant in Kruskal–Wallis ANOVA (*p* < 0.05), while the differences in concentrations of the analyzed macronutrients (Figure 2) were irrelevant (*p* > 0.05). Energy of breast milk in the study groups was as follows: median (IQR) 55.5 (54.0–68.0) kcal/dl in GDM G1, median (IQR) 55.5 (50.0–64.5) kcal/dL in GDM G2, and median (IQR) 65.0 (53.5–77.0) kcal/dL in non-GDM. Thus, mothers in the control group produced the most caloric milk in the first week postpartum. Furthermore, the narrowest range of caloric values was observed in this group.

### 3.3. Cluster Analysis

Four patient clusters were distinguished based on the following factors: belonging to a particular study group, maternal history of hypothyroidism, and weight gain during pregnancy. The ‘Cluster 1’ consisted of mothers diagnosed with gestational diabetes who did not exhibit any thyroid dysfunction. The ‘Cluster 2’ comprised mothers who had been diagnosed with both gestational diabetes and hypothyroidism. The ‘Cluster 3’ comprised newborns of healthy mothers, free of any diabetic or thyroid disorders. The ‘Cluster 4’ consisted of non-diabetic mothers with concomitant hypothyroidism. The highest maternal weight gain was observed in ‘Cluster 3’. The methodology employed for cluster identification has been previously described in detail elsewhere [14]. The macronutrient composition of maternal breast milk was compared between the patient clusters. Only true protein concentrations were found to be significantly different (*p* < 0.05), with the highest values in Cluster 3 and the lowest in Cluster 4. With regard to these findings, maternal history of hypothyroidism was associated with true protein content among mothers without impaired glucose metabolism. However, this effect was not observed among mothers with GDM. The concentrations of the other macronutrients (fat, total protein, carbohydrates, dry matter) and energy were statistically similar (*p* > 0.05). The detailed results of the comparisons between the clusters are shown in Table 1.

### 3.4. Influence of Neonatal Birth Weight (BW) and Body Fat Mass Percent (%FBM) on Maternal Milk Composition

The results of the regression analysis are summarized in the table below (Table 2). Regarding birth weight, there was no significant association with the macronutrient composition of breast milk in the first week postpartum (*p* > 0.05). In terms of percent body fat mass, a significant relationship was found with concentrations of total protein, true protein, and dry matter (*p* < 0.05). The concentrations of fat and carbohydrate as well as energy were not related to either the BW or the %FBM (*p* > 0.05).

### 3.5. Other Maternal Factors

In addition to classification by study group, maternal history of hypothyroidism and maternal weight gain during pregnancy were selected as the best predictors of neonatal anthropometrics and body composition, while defining clusters [14]. Based on the medical interview, mothers were classified by history of hypothyroidism (gestational, chronic, none), BMI prior to the pregnancy (normal, overweight, obese—none of the enrolled mothers were found to be underweight), weight gain during pregnancy in reference to pre-gestational BMI (recommendations below, within recommendations, above recommendations). Accordingly, breast milk composition was compared between mothers. The only significant (*p* < 0.05) difference was found in true protein concentrations according to the classification of hypothyroidism history in the maternal medical interview. Mothers with no history of hypothyroidism were found to have the highest true protein concentrations. The detailed results are presented in Table 3.

## 4. Discussion

In our study, the analysis of breast milk collected during the first week postpartum yielded only one statistically significant result, namely a difference in energy content between the study groups. The milk produced by the mothers in the control group (non-GDM) was found to have the highest caloric value. In addition, this group was also found to be characterized by the narrowest range of energy values. As previously reported, these mothers exhibited the highest gestational weight gain (GWG), which frequently exceeded the obstetric recommendations for pre-pregnancy BMI [14]. Conversely, these mothers exhibited the lowest mean BMI prior to the pregnancy and a reduced prevalence of other concomitant disorders (e.g., hypertension, hypothyroidism, obesity) compared to GDM mothers. The authors hypothesize that these factors may influence the impact of GWG and maternal general genetic conditioning on breast milk production. Mothers with gestational diabetes treated with dietary intervention (GDM G1) had similar milk composition to the mothers with insulin-treated gestational diabetes (GDM G2). This might be the result of good glycemic control in both groups, even though they differed in the severity of glucose intolerance.

When analyzed in clusters, the significant difference concerned true protein. The highest concentrations were identified in Cluster 3, which included non-GDM mothers with no history of hypothyroidism but high GWG. In contrast, the lowest concentrations were observed in Cluster 4, which comprised non-GDM mothers who were diagnosed with hypothyroidism and also exhibited higher GWG than diabetic mothers. It is therefore hypothesized that thyroid function may influence the true protein content of human milk. Our findings appear to align with those of a systematic review conducted by Lopes et al. The included studies indicated a reduction in protein concentration in breast milk during the first four weeks postpartum, with an average decline in milk protein concentration among mothers with thyroid disease [6].

The composition of breast milk has been found to be influenced by a number of maternal factors. With regard to macronutrients, there is evidence that their concentrations are significantly influenced by maternal BMI and gestational weight gain, particularly in terms of fat and protein content [17,18,19], but also energy [18]. The majority of these differences were documented in studies on mature milk (at the four-week mark), with no discernible alterations in its overall composition over the initial four-week period [18]. The available evidence is limited with regard to the modulation of breast milk composition over time [17]. However, the concentration of fat in HM is known to exhibit significant fluctuations, both diurnal and inter-feed. Furthermore, the substantial heterogeneity of results between studies and the low quality of many of them should be taken into account [19]. Also, the conclusions regarding how gestational diabetes affects breast milk composition are not entirely conclusive, given the limited number of studies and the variability in the design and implementation of their methods. Overall, it can be posited that GDM may alter the composition of human milk [5,20]. Furthermore, Peila et al. determined that diabetes (both GDM and pregestational/overt diabetes) can impact the composition of human milk [21]. However, the systematic review revealed several limitations. These included the small number of available studies, the referencing of different stages of lactation, the use of various definitions of human milk types, and the timing of sample collection. Furthermore, there was a lack of information on maternal treatment. Additionally, the reviewed studies reported inconsistent findings with regard to selected macronutrients [21]. The results of our study may differ from those of the aforementioned review, as our analysis was limited to hindmilk collected during the first week postpartum from women with other concomitant diseases.

The heterogeneity of human milk composition appears to be a pivotal factor in the nutritional programming of breastfed infants [5,20,22]. In the study by de Fluiter et al., long-term changes in macronutrient composition, particularly fat and energy in breast milk, were associated with infant weight gain, especially fat body mass [23]. In addition, the fatty acid profile of breast milk, which depends to some extent on the mother’s diet as well as maternal weight and BMI, appears to be related to infant weight, BMI, lean body mass (LBM), and fat mass (FBM) [24,25]. Based on the review by Rice and Valentine, higher protein intake results in higher LBM growth, greater increase in head circumference, and better brain growth, and that growth rate was correlated with neurodevelopmental and mental outcomes. Increase in non-protein energy intake resulted in higher FBM [26].

In our study, regression analysis resulted in a weak, but significant relationship between neonatal %FBM and total protein, true protein, and dry matter in maternal milk. Further research is needed to explain this relationship and verify the correlation between neonatal body composition and maternal milk composition when analyzing their changes in time. In fact, in our study, the obtained results seem to be highly affected by maternal weight gain in pregnancy. As previously reported [14], newborns of non-GDM mothers were found to have the highest birth weight and the lowest percentage of lean body mass in the first week after birth, but these results were not significant. In addition, infants born to mothers with well-controlled GDM and hypothyroidism had significantly lower birth weight and %FBM than infants born to generally healthy mothers, but in contrast to the control group, the GDM group also had well-controlled body weight and GWG [14]. In our opinion, regular medical surveillance led to more effective control of gestational weight gain, which may explain the results obtained.

The main limitation of the current study is the lack of possibility to analyze breast milk samples collected several times consecutively in the first week postpartum due to lack of maternal consent. Another limitation is the lack of follow-up for further breast milk analysis—mothers stopped breastfeeding or were unwilling to participate in follow-up. Unfortunately, the mothers who accepted the invitation to participate in the study had several concomitant factors in their interviews, so it was not possible to distinguish clear groups with only one factor, which would have given us clear results.

In our study, we employed the cluster analysis method, which permitted the comparison of results between groups of patients with consideration of more than one characteristic. In our case, these were three factors: GDM, hypothyroidism, and weight gain. By utilizing this non-standardized method, we demonstrated that discrepancies in results may be attributed to the cumulative effect of multiple factors, rather than to a single primary disease. This approach is one of the major strengths of our study. We would like to emphasize that the use of the above-mentioned method, allows to distinguish and compare clinical groups of patients, in whom the metabolic profile may depend on several factors. The cumulative effect of comorbidities in pregnant women can have an impact on pregnancy outcomes, including neonatal outcomes and breast milk composition. We believe that a comprehensive assessment of the profile of patients can be important in evaluating the effects of treatment and modifying the therapeutic plan, also in terms of perinatal and neonatal care.

Another strength of our work is the comprehensive assessment of factors that can affect the composition of breast milk. In addition to the mother’s medical history, we considered the course of pregnancy and neonatal factors—birth weight and precent fat body mass. Further research is required to ascertain the long-term relationship between human milk components and neonatal anthropometrics.

## 5. Conclusions

Breast milk composition in the first week after birth is influenced by maternal health status, as well as maternal body weight, BMI, and gestational weight gain. Further research is needed to verify the relationship between breast milk macronutrients and neonatal body composition in the first week of life, as well as their changes over time. Further research is needed to determine the significance of the differing macronutrient composition of breast milk in early infancy.

## Figures and Tables

**Table 1:** Breast milk macronutrient composition in clusters.

Macronutrients (g/dL; # Energy kcal/dL)	All Participants (*n* = 70)	Cluster 1 (*n* = 23)	Cluster 2 (*n* = 27)	Cluster 3 (*n* = 14)	Cluster 4 (*n* = 6)	*p* Value
Fat (Mean, SD)	2.2, 1.02	2.2, 0.9	2.1, 1.2	2.2, 0.7	2.6, 0.9	0.762
Total protein (Median, IQR)	2.1, 1.8–2.5	2.1, 1.9–2.8	2.1, 1.8–2.5	2.4, 2.1–4.4	1.8, 1.7–1.9	0.051
Carbohydrates (Mean, SD)	7.2, 0.82	7.2, 1.1	7.2, 0.7	7.4, 0.3	7.3, 1.1	0.785
Dry matter (Median, IQR)	12.0, 11.2–13.3	11.8, 11.3–12.9	11.6, 10.7–13.3	12.0, 11.8–14.0	12.7, 11.6–13.3	0.089
Energy (Mean, SD) ^#^	60.3, 9.6	59.4, 8.3	58.4, 1.4	63.4, 7.4	64.9, 8.0	0.246
True protein (Median, IQR)	1.7, 1.5–2.1	1.7, 1.5–2.0	1.7, 1.4–2.1	2.0, 1.7–3.15	1.45, 1.4–1.5	0.041 *

* statistically significant for *p* < 0.05; # refers to results concerning energy.

**Table 2:** Prediction of macronutrient content in maternal milk in the first week postpartum by birth weight (BW) and percent of neonatal fat body mass (%FBM).

	BW			%FBM		
Macronutrient	Coefficient	95% CI	*p* Value	Coefficient	95% CI	*p* Value
Fat	−0.090	−0.590, 0.410	0.725	0.035	−0.068, 0.138	0.508
Total protein	0.4234	−0.035, 0.883	0.075	0.113	0.020, 0.206	0.020 *
Carbohydrates	0.348	−0.045, 0.741	0.880	−0.036	−0.118, 0.046	0.396
Dry matter	0.445	−0.380, 0.869	0.245	0.128	0.003, 0.254	0.048 *
Energy	2.244	−2.424, 6.911	0.249	0.925	−0.017, 1.867	0.060
True protein	0.256	−0.128, 0.639	0.196	0.103	0.027, 0.179	0.010 *

* statistically significant for *p* < 0.05.

**Table 3:** Breast milk composition in the first week after delivery by maternal factors.

Macronutrient [g/dL; ^#^ Energy kcal/dL]	Maternal Factor			
	History of Hypothyroidism			
	Gestational (with Onset during Pregnancy) (*n* = 13)	Chronic (with Onset Before Pregnancy) (*n* = 20)	None (*n* = 37)	*p* Value
Fat	2.3, 1.3–2.8	1.5, 1.2–3.5	2.1, 1.6–2.7	0.665
Total protein	2.1, 1.9–2.5	1.9, 1.8–2.3	2.2, 1.9–2.8	0.062
Carbohydrates	7.4, 7.1–7.7	7.4, 7.2–7.5	7.5, 7.2–7.6	0.769
Dry matter	12.6, 11.6–12.9	11.3, 10.8–13.4	12.0, 11.6–12.9	0.364
Energy ^#^	60.5, 55.0–67.5	55.0, 48.5–68.5	58.0, 55.0–67.5	0.308
True protein	1.7, 1.5–2.6	1.5, 1.4–1.8	1.8, 1.5–2.3	0.024 *
	Pre-pregnancy BMI [kg/m^2^]			
	Normal (*n* = 40)	Overweight (*n* = 16)	Obese (*n* = 14)	*p* value
Fat	2.1, 1.6–2.8	2.0, 1.4–3.3	1.5, 1.2–2.4	0.269
Total protein	2.1, 1.8–2.6	2.1, 1.8–2.2	2.4, 2.1–2.7	0.121
Carbohydrates	7.4, 7.1–7.6	7.5, 7.3–7.6	7.5, 7.3–7.6	0.699
Dry matter	12.3, 11.6–13.1	11.7, 10.6–13.2	12.1, 10.9–14.0	0.459
Energy ^#^	57.5, 55.0–67.0	56.8, 50.0–71.7	59.3, 50.0–66.0	0.752
True protein	1.7, 1.4–2.3	1.7, 1.5–1.8	1.9, 1.7–3.1	0.086
	Gestational weight gain in reference to pre-gestational BMI			
	Below recommendations (*n* = 18)	Within recommendations (*n* = 26)	Above recommendations (*n* = 26)	*p* value
Fat	1.9, 1.3–3.3	1.8, 1.3–2.9	2.1, 1.6–2.8	0.799
Total protein	2.1, 1.9–2.6	2.0, 1.8–2.3	2.2, 1.8–2.7	0.377
Carbohydrates	7.5, 7.3–7.6	7.4, 7.2–7.6	7.5, 7.1–7.7	0.690
Dry matter	12.3, 11.5–13.3	11.6, 10.9–12.6	12.6, 11.7–13.5	0.133
Energy	57.0, 54.0–68.0	55.5, 51.0–65.0	62.8, 55.5–68.0	0.241
True protein	1.7, 1.6–2.6	1.6, 1.4–2.0	1.8, 1.5–2.1	0.456

Data are presented as median with interquartile range (IQR). The *p* value < 0.05 was considered statistically significant. * statistically significant for *p* < 0.05; # refers to results concerning energy.

## Data Availability

Anonymized aggregate data are available from the corresponding author upon reasonable request and in accordance with authors’ institutional policy and restrictions outlined in participant consent agreements.

## References

[1] Kim S.Y., Yi D.Y. (2020). Components of human breast milk: From macronutrient to microbiome and microRNA. Clin. Exp. Pediatr..

[2] Golan Y., Assaraf Y.G. (2020). Genetic and Physiological Factors Affecting Human Milk Production and Composition. Nutrients.

[3] Institute of Medicine (US) Committee on Nutritional Status During Pregnancy and Lactation (1991). Nutrition During Lactation.

[4] Samuel T.M., Zhou Q., Giuffrida F., Munblit D., Verhasselt V., Thakkar S.K. (2020). Nutritional and Non-nutritional Composition of Human Milk Is Modulated by Maternal, Infant, and Methodological Factors. Front. Nutr..

[5] Wu Y. (2023). The Impact of Gestational Diabetes Mellitus on the Composition of Breast Milk: A Systematic Review. HSET.

[6] Lopes F.O., Soares F.V.M., Silva D.A.D., Moreira M.E.L. (2020). Do Thyroid Diseases during Pregnancy and Lactation Affect the Nutritional Composition of Human Milk? As doenças da tireoide durante a gestação e lactação afetam a composição nutricional do leite humano?. Rev. Bras. Ginecol. Obstet..

[7] Hashemi Javaheri F.S., Karbin K., Senobari M.A., Hakim H.G., Hashemi M. (2024). The association between maternal body mass index and breast milk composition: A systematic review. Nutr. Rev..

[8] Meek J.Y., Noble L. (2022). Section on Breastfeeding; Policy Statement: Breastfeeding and the Use of Human Milk. Pediatrics.

[9] Horta B.L., Victora C.G., World Health Organization Long-Term Effects of Breastfeeding: A Systematic Review. http://www.who.int/iris/handle/10665/79198.

[10] (2018). Optimizing support for breastfeeding as part of obstetric practice. ACOG Committee Opinion No. 756. American College of Obstetricians and Gynecologists. Obstet. Gynecol..

[11] Nommsen L.A., Lovelady C.A., Heinig M.J., Lönnerdal B., Dewey K.G. (1991). Determinants of energy, protein, lipid, and lactose concentrations in human milk during the first 12 months of lactation: The DARLING Study. Am. J. Clin. Nutr..

[12] Lagström H., Rautava S., Ollila H., Kaljonen A., Turta O., Mäkelä J., Yonemitsu C., Gupta J., Bode L. (2020). Associations between human milk oligosaccharides and growth in infancy and early childhood. Am. J. Clin. Nutr..

[13] Butte N.F., Garza C., Smith E.O., Nichols B.L. (1984). Human milk intake and growth in exclusively breast-fed infants. J. Pediatr..

[14] Karcz K., Czosnykowska-Lukacka M., Krolak-Olejnik B. (2023). Impact of gestational diabetes and other maternal factors on neonatal body composition in the first week of life: A case-control study. Ginekol. Pol..

[15] Marczewski E., Steinhaus H. (1958). On a certain distance of sets and the corresponding distance of functions. Colloq. Math..

[16] Zhai C.X. (2007). A Note on the Expectation-Maximization (EM) Algorithm.

[17] Sims C.R., Lipsmeyer M.E., Turner D.E., Andres A. (2020). Human milk composition differs by maternal BMI in the first 9 months postpartum. Am. J. Clin. Nutr..

[18] Borràs-Novell C., Herranz Barbero A., Balcells Esponera C., López-Abad M., Aldecoa Bilbao V., Izquierdo Renau M., Iglesias Platas I. (2023). Influence of maternal and perinatal factors on macronutrient content of very preterm human milk during the first weeks after birth. J. Perinatol..

[19] Leghi G.E., Netting M.J., Middleton P.F., Wlodek M.E., Geddes D.T., Muhlhausler A.B.S. (2020). The impact of maternal obesity on human milk macronutrient composition: A systematic review and meta-analysis. Nutrients.

[20] Dugas C., Laberee L., Perron J., St-Arnaud G., Richard V., Perreault V., Leblanc N., Marc I., Di Marzo V., Doyen A. (2023). Gestational Diabetes Mellitus, Human Milk Composition, and Infant Growth. Breastfeed. Med..

[21] Peila C., Gazzolo D., Bertino E., Cresi F., Coscia A. (2020). Influence of Diabetes during Pregnancy on Human Milk Composition. Nutrients.

[22] Şahin Ö.N., Di Renzo G.C., Şahin Ö.N., Briana D.D., Di Renzo G.C. (2023). Gestational Diabetes and Variety in the Composition of Breast Milk. Breastfeeding and Metabolic Programming.

[23] de Fluiter K.S., Kerkhof G.F., van Beijsterveldt I.A.L.P., Breij L.M., van de Heijning B.J.M., Abrahamse-Berkeveld M., Hokken-Koelega A.C.S. (2021). Longitudinal human milk macronutrients, body composition and infant appetite during early life. Clin. Nutr..

[24] Much D., Brunner S., Vollhardt C., Schmid D., Sedlmeier E.M., Brüderl M., Heimberg E., Bartke N., Boehm G., Bader B.L. (2013). Breast milk fatty acid profile in relation to infant growth and body composition: Results from the INFAT study. Pediatr. Res..

[25] Simon Sarkadi L., Zhang M., Muránszky G., Vass R.A., Matsyura O., Benes E., Vari S.G. (2022). Fatty Acid Composition of Milk from Mothers with Normal Weight, Obesity, or Gestational Diabetes. Life.

[26] Rice M.S., Valentine C.J. (2015). Neonatal Body Composition: Measuring Lean Mass as a Tool to Guide Nutrition Management in the Neonate. Nutr. Clin. Pract..

